# Downregulation of osteoprotegerin expression in metastatic colorectal carcinoma predicts recurrent metastasis and poor prognosis

**DOI:** 10.18632/oncotarget.12686

**Published:** 2016-10-15

**Authors:** Ahrim Moon, Sung-Im Do, Hyun-Soo Kim, Youn-Wha Kim

**Affiliations:** ^1^ Department of Pathology, Soonchunhyang University Bucheon Hospital, Soonchunhyang University College of Medicine, Bucheon-si, Gyeonggi-do, Republic of Korea; ^2^ Department of Pathology, Kangbuk Samsung Hospital, Sungkyunkwan University School of Medicine, Seoul, Republic of Korea; ^3^ Department of Pathology, Severance Hospital, Yonsei University College of Medicine, Seoul, Republic of Korea; ^4^ Department of Pathology, Kyung Hee University School of Medicine, Seoul, Republic of Korea

**Keywords:** osteoprotegerin, downregulation, colorectal carcinoma, recurrent metastasis, immunohistochemistry

## Abstract

We recently reported the downregulation of osteoprotegerin expression in primary colorectal carcinoma and its significant association with aggressive oncogenic behavior, which suggest that this process contributes to colorectal carcinoma development and progression. In this study, we used immunohistochemical staining to evaluate osteoprotegerin expression in 81 colorectal liver metastasis tissue samples and investigated its possible association with the clinicopathological characteristics and outcomes of patients with colorectal liver metastasis. These tissues exhibited significantly reduced expression of osteoprotegerin compared to primary colorectal carcinomas and normal colorectal mucosa. This reduced expression was significantly associated with the extent of colorectal liver metastasis, including multiplicity of metastatic tumors, involvement of the bilateral hepatic lobes, and higher histological grade. In addition, reduced osteoprotegerin expression was an independent significant predictor of recurrent liver metastasis and prognostic factor for reduced patient survival. These findings suggest that osteoprotegerin expression may be a novel predictor of recurrent liver metastasis and a prognostic biomarker in patients with colorectal liver metastasis. Patients harboring colorectal liver metastasis with reduced osteoprotegerin expression should be carefully monitored after hepatic resection for colorectal liver metastasis to enable early detection of potentially resectable metastatic recurrences.

## INTRODUCTION

Colorectal carcinoma is the fourth most common cancer in Western countries and the second leading cause of cancer-related deaths in Europe and North America [1, 2]. In Korea, colorectal carcinoma is the third most common cancer, after thyroid carcinoma and stomach carcinoma, and is the fourth leading cause of cancer-related deaths [3]. Despite major advances in surgical techniques and chemotherapeutic agents, the prognosis of colorectal carcinoma patients remains poor due to development of distant metastasis and recurrence [4]. The liver is the most common site of metastasis for colorectal carcinoma; more than 50% of patients with primary colorectal carcinoma will develop liver metastasis during their lifetimes [1, 2, 5]. Untreated colorectal liver metastasis has poor prognosis with a median survival of 6-12 months [4]. Evidence from numerous retrospective and comparative studies indicates that hepatic resection is the best potential curative treatment for colorectal liver metastasis that promotes long-term survival [6]. Hepatic resection for colorectal liver metastasis is associated with a 5-year survival rate of 25–51% [7].

Identification of patients at high risk for liver metastasis is essential to determine candidates for hepatic resection but has previously relied on assessment of conventional pathological characteristics of colorectal carcinoma, including invasion depth, lymph node metastasis, and stage. However, the current tumor-node-metastasis staging system is limited, as it cannot offer a prognosis for individual patients [4]. Emerging strategies designed to increase the proportion of patients who are candidates for hepatic resection have been introduced in clinical practice, but most patients with colorectal liver metastasis are not candidates for this procedure [2]. To improve the outcome of patients with colorectal carcinoma with or without subsequent liver metastasis, identifying cancer-related genes as predictive and prognostic biomarkers for personalized therapy is critical.

Osteoprotegerin, a secreted member of the tumor necrosis factor receptor superfamily, has many biological functions, such as a potent inhibitor of osteoclastic bone resorption and a potential therapeutic agent for treatment of osteoporosis [8]. In addition to its role in bone metabolism, osteoprotegerin is involved in the development and progression of several human malignancies [4, 9–16]. We recently reported that osteoprotegerin expression is downregulated in primary colorectal carcinoma cell lines and tissues. This reduction is significantly associated with aggressive oncogenic behavior of colorectal carcinoma, including higher histological grade, lymph node metastasis, liver metastasis, advanced stage, and vascular invasion [4]. Furthermore, osteoprotegerin expression is an independent predictor of reduced survival of colorectal carcinoma patients [4].

Existing evidence for osteoprotegerin's important role in tumor progression and metastasis of colorectal carcinoma prompted us to examine its expression in colorectal liver metastasis tissue samples and to investigate its potential relationship with clinicopathological characteristics and clinical outcomes of patients with colorectal liver metastasis. As a result, we identified independent predictors for recurrent liver metastasis that could facilitate the identification of patients at increased risk for developing metastatic recurrence.

## RESULTS

### Patient demographics

Primary colorectal carcinoma tissue and the corresponding colorectal liver metastasis tissue samples were obtained from 81 patients. The median patient age was 60 years (range: 29–76 years), and 51.9% (42/81) of patients were aged 60 years or older. The study group included 56 males and 25 females. Numbers of metastatic tumors per patient were as follows: 1 in 56.8% (46/81), 2 in 22.2% (18/81), 3 in 8.6% (7/81), and 4 or more in 12.3% (10/81) of patients. The median size of metastatic tumors was 2.5 cm; 46 (56.8%) patients had metastatic tumors with a maximum diameter of 2.5 cm or greater. Histological grades of primary colorectal carcinoma were 1 (well-differentiated) in 17.3% (14/81), 2 (moderately-differentiated) in 79.0% (64/81), and 3 (poorly-differentiated) in 3.7% (3/81) of patients. The microsatellite instability (MSI) status of primary colorectal carcinoma was available in 84.0% (68/81) of patients and revealed that 6 (8.8%) of these 68 patients had MSI. Forty-nine (60.5%) patients developed recurrent liver metastasis after hepatic resection.

### Downregulation of osteoprotegerin expression in primary colorectal carcinoma and colorectal liver metastasis

**Figure 1:**
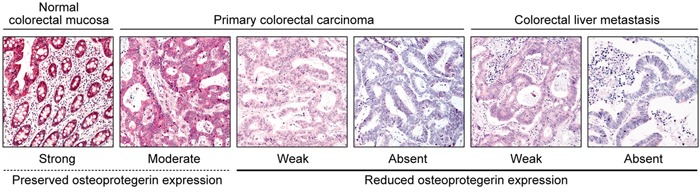
Osteoprotegerin immunoreactivity in normal colorectal mucosa, primary colorectal carcinoma, and colorectal liver metastasis Representative photomicrographs are shown. Original magnification, 100×.

We measured osteoprotegerin expression in normal colorectal mucosa, primary colorectal carcinomas, and colorectal liver metastasis tissue samples using immunohistochemistry. Osteoprotegerin immunoreactivity was predominantly cytoplasmic, although weak nuclear staining was noted in some tumor cells. In a few cases that showed strong osteoprotegerin expression in tumor cells, faint osteoprotegerin expression was noted in the extracellular matrix or connective tissue. Representative photomicrographs of osteoprotegerin immunostaining in normal colorectal mucosa, primary colorectal carcinomas, and colorectal liver metastasis tissues are shown in Figure 1. In 18 normal colorectal mucosal tissue samples, strong, uniform osteoprotegerin immunoreactivity was observed in the cytoplasm of epithelial cells. Among the primary colorectal carcinoma tissues, reduced osteoprotegerin expression was observed in 61.7% (50/81) of tumor tissue samples. Osteoprotegerin immunoreactivity was moderate in 38.3% (31/81), weak in 32.1% (26/81), and absent in 29.6% (24/81) of primary colorectal carcinoma samples. Reduced osteoprotegerin expression was observed in 75.3% (61/81) of metastatic tissue samples. Osteoprotegerin immunoreactivity was moderate in 24.7% (20/81), weak in 32.1% (26/81), and absent in 43.2% (35/81) of colorectal liver metastasis samples. Osteoprotegerin expression in colorectal liver metastases was significantly decreased compared to that in primary colorectal carcinoma tissue samples (*P*=0.036; Table 1).

**Table 1:** Differences in osteoprotegerin expression between primary colorectal carcinoma and colorectal liver metastasis

Category	Number of cases (%)					*P* value
	Total	Osteoprotegerin expression status				
		Reduced		Preserved		
		0	1+	2+	3+	
Colorectal liver metastasis	81	35 (43.2)	26 (32.1)	20 (24.7)	0 (0.0)	0.036*
Primary colorectal carcinoma	81	24 (29.6)	26 (32.1)	31 (38.3)	0 (0.0)	

* Statistically significant.

### Relationship of osteoprotegerin immunoreactivity with the clinicopathological characteristics of colorectal liver metastasis

The association between osteoprotegerin expression and the clinicopathological characteristics of colorectal liver metastasis patients is illustrated in Table 2. We observed significant inverse relationships between osteoprotegerin expression and multiplicity of metastatic tumors (*P*<0.001), bilobar involvement of metastatic tumors (*P*=0.046), higher histological grade of primary colorectal carcinoma (*P*<0.001), and occurrence of recurrent liver metastasis (*P*<0.001).

**Table 2:** Relationship between osteoprotegerin expression and patient clinicopathological characteristics

Characteristics		Number of cases (%)			*P* value
		Total	Osteoprotegerin expression status		
			Reduced	Preserved	
Age (years)	≥60	42	31 (73.8)	11 (26.2)	0.745
	<60	39	30 (76.9)	9 (23.1)	
Sex	Male	56	41 (73.2)	15 (26.8)	0.513
	Female	25	20 (80.0)	5 (20.0)	
Number of metastatic tumor	Multiple	35	34 (97.1)	1 (2.9)	<0.001*
	Single	46	27 (58.7)	19 (41.3)	
Size of metastatic tumor (cm)	≥2.5	46	34 (73.9)	12 (26.1)	0.738
	<2.5	35	27 (77.1)	8 (22.9)	
Distribution of metastatic tumor	Bilobar	23	22 (95.7)	1 (4.3)	0.009*
	Unilobar	58	39 (67.2)	19 (32.8)	
Histological grade of primary tumor	3	3	3 (100.0)	0 (0.0)	<0.001*
	2	64	54 (84.4)	10 (15.6)	
	1	14	4 (28.6)	10 (71.4)	
Microsatellite instability status	MSI	6	3 (50.0)	3 (50.0)	0.338
	MSS	62	46 (74.2)	16 (25.8)	
Recurrent liver metastasis	Present	49	45 (91.8)	4 (8.2)	<0.001*
	Absent	32	16 (50.0)	16 (50.0)	

Abbreviations: MSI: microsatellite instability, MSS: microsatellite stable;

* Statistically significant.

### Predictive value of osteoprotegerin expression for recurrent liver metastasis

Multiplicity of metastatic tumors (*P*<0.001), bilobar involvement of metastatic tumors (*P*=0.007), and reduced osteoprotegerin expression (*P*<0.001) were significantly associated with the recurrence of colorectal liver metastasis (Table 3). To identify the independent predictive factors for recurrent liver metastasis development, these three covariates were included in a multivariate logistic regression analysis. Multiplicity of metastatic tumors (*P*=0.002; relative risk=7.145; 95% confidence interval=2.015-25.337) and reduced osteoprotegerin expression (*P*=0.012; relative risk=5.425; 95% confidence interval=1.443-20.397) were independently predicted recurrent liver metastasis in our study group.

**Table 3:** Factors predicting recurrent liver metastasis

Characteristics		Univariate				Multivariate	
		Number of cases (%)			*P* value	Relative risk (95% confidence interval)	*P* value
		Total	Recurrent liver metastasis				
			Present	Absent			
Age (years)	≥60	42	22 (52.4)	20 (47.6)	0.121	Not applicable	
	<60	39	27 (69.2)	12 (30.8)			
Sex	Male	56	35 (62.5)	21 (37.5)	0.580	Not applicable	
	Female	25	14 (56.0)	11 (44.0)			
Number of metastatic tumor	Multiple	35	31 (88.6)	4 (11.4)	<0.001*	7.145 (2.015–25.337)	0.002*
	Single	46	18 (39.1)	28 (60.9)			
Size of metastatic tumor (cm)	≥2.5	46	30 (65.2)	16 (34.8)	0.319	Not applicable	
	<2.5	35	19 (54.3)	16 (45.7)			
Distribution of metastatic tumor	Bilobar	23	20 (87.0)	3 (13.0)	0.002*	0.683 (0.062–7.482)	0.683
	Unilobar	58	29 (50.0)	29 (50.0)			
Histological grade of primary tumor	3	3	2 (66.7)	1 (33.3)	0.059	Not applicable	
	2	64	42 (65.6)	22 (34.4)			
	1	14	5 (35.7)	9 (64.3)			
Microsatellite instability status	MSI	6	2 (33.3)	4 (66.7)	0.390	Not applicable	
	MSS	62	37 (59.7)	25 (40.3)			
Osteoprotegerin expression status	Reduced	61	45 (73.8)	16 (26.2)	<0.001*	5.425 (1.443–20.397)	0.012*
	Preserved	20	4 (20.0)	16 (80.0)			

Abbreviations: MSI: microsatellite instability, MSS: microsatellite stable;

* Statistically significant.

### Prognostic significance of osteoprotegerin expression for colorectal liver metastasis

**Figure 2:**
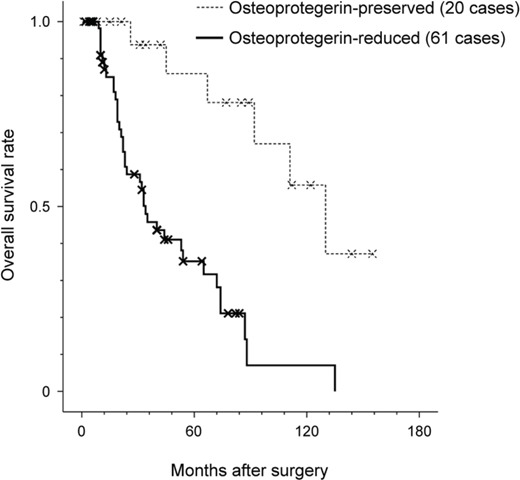
Prognostic significance of osteoprotegerin expression in colorectal liver metastasis The Kaplan-Meier plot of overall survival of patients with colorectal liver metastasis. Reduced osteoprotegerin expression was significantly associated with reduced overall survival (*P*<0.001). The median survival duration of patients with reduced osteoprotegerin expression was 34 months compared to 92 months in patients with preserved osteoprotegerin expression. Log-rank test.

The prognostic value of osteoprotegerin expression for hepatic colorectal metastasis was evaluated. Complete data were available for all 81 patients. Forty-nine (60.5%) patients died prior to their final follow-up visit. A univariate survival analysis showed that the multiplicity of metastatic tumors (*P*<0.001), bilobar involvement of metastatic tumors (*P*=0.002), recurrent liver metastasis (*P*<0.001), and reduced osteoprotegerin expression (*P*<0.001) were significant predictors of poor prognosis (Table 4). The median survival duration of patients whose tumors exhibited reduced osteoprotegerin expression was 34 months compared to 92 months in patients with preserved osteoprotegerin expression (Figure 2). Patients with osteoprotegerin-preserved tumors had stable survival rates of 93.6% and 79.3% at 3 and 5 years post-surgery, respectively, as shown by a Kaplan-Meier plot. In contrast, patients with reduced osteoprotegerin expression experienced a rapid decline in survival during the observation period. Survival rates were 43.6% at 3 years and 27.1% at 5 years post-surgery in patients with osteoprotegerin-reduced colorectal liver metastasis.

**Table 4:** Factors predicting reduced overall patient survival

Characteristics		Univariate	Multivariate	
		*P* value	Hazard ratio (95% confidence interval)	*P* value
Age (years)	≥60/<60	0.620	Not applicable	
Sex	Male/Female	0.399	Not applicable	
Number of metastatic tumor	Multiple/Single	<0.001*	0.865 (0.356–2.104)	0.749
Size of metastatic tumor (cm)	≥2.5/<2.5	0.661	Not applicable	
Distribution of metastatic tumor	Bilobar/Unilobar	0.026*	1.595 (0.848–3.002)	0.162
Histological grade of primary tumor	2–3/1	0.470	Not applicable	
Microsatellite instability status	MSI/MSS	0.536	Not applicable	
Recurrent liver metastasis	Present/Absent	<0.001*	2.401 (1.203–4.794)	0.013*
Osteoprotegerin expression status	Reduced/Preserved	<0.001*	2.894 (1.272–6.585)	0.011*

Abbreviations: MSI: microsatellite instability, MSS: microsatellite stable;

* Statistically significant.

We also conducted multivariate survival analysis using a Cox proportional hazard regression model. Osteoprotegerin expression (*P*=0.011; hazard ratio=2.894; 95% confidence interval=1.272–6.585) and recurrent liver metastasis (*P*=0.013; hazard ratio=2.401; 95% confidence interval=1.203–4.794) were independent prognostic factors influencing overall survival (Table 4). This analysis demonstrated that reduced osteoprotegerin expression was associated with a significant hazard ratio of 2.894, which was greater than the risk associated with recurrent liver metastasis (2.401).

## DISCUSSION

We determined that colorectal liver metastasis tissues exhibit significantly reduced osteoprotegerin expression compared to primary colorectal carcinomas and normal colorectal mucosa tissues. This finding is consistent with our recent observation of significantly decreased osteoprotegerin expression in 68.4% of primary colorectal carcinoma cases but none in normal colorectal mucosa [4]. The frequency of reduced osteoprotegerin expression in primary colorectal carcinomas was similar to that demonstrated in our previous study. We also saw a significant association between reduced osteoprotegerin expression and the multiplicity and bilobar involvement of metastatic tumors and the occurrence of recurrent liver metastasis, which corroborates our previous findings that reduced osteoprotegerin expression is significantly associated with aggressive oncogenic behavior in primary colorectal carcinoma, including larger tumor size, higher histological grade, lymph node metastasis, colorectal liver metastasis, advanced stage, and vascular invasion [4]. Taken together, our data indicate that reduced osteoprotegerin expression is involved in the development, progression, and metastasis of colorectal carcinoma, suggesting that osteoprotegerin functions as a tumor suppressor in colorectal carcinoma. To confirm these findings, it will be necessary to analyze osteoprotegerin expression using a larger number of primary and metastatic colorectal carcinoma tissue samples.

Furthermore, we demonstrated that reduced osteoprotegerin expression in colorectal liver metastasis predicts the occurrence of recurrent liver metastasis. While hepatic resection is the gold standard treatment for colorectal liver metastasis and is a safe procedure [17], approximately 60-70% of patients undergoing this procedure experience disease recurrence, often in the first 12-18 postoperative months [18]. Of these patients, one-third will have recurrent metastases restricted to the liver. Because hepatic resection has become safer through improvements in surgical techniques and perioperative management, repeat hepatic resection is performed more frequently in patients with recurrent liver metastasis [19], which may be warranted due to the procedure's expected outcomes that rival those of single hepatic resection [20]. Therefore, developing biomarker-based criteria to enable earlier and improved stratification of patients according to their risk of recurrence and survival and consequent selection of patients who may benefit from repeat hepatic resection is needed [7]. Our findings indicate that osteoprotegerin expression in colorectal liver metastasis tissues may be such a biomarker, as reduced osteoprotegerin expression is a significant independent predictor of recurrent liver metastasis. Our data suggest that patients harboring colorectal liver metastasis with reduced osteoprotegerin expression require careful monitoring after hepatic resection for colorectal metastasis to facilitate early detection of potentially resectable metastatic recurrences.

We observed that reduced osteoprotegerin expression is significantly associated with reduced overall survival in patients with colorectal liver metastasis. This relationship persisted after adjusting for other significant clinicopathological characteristics in the multivariate analysis, indicating that reduced osteoprotegerin expression is an independent prognostic factor for lower survival rates in patients with colorectal liver metastasis. Similarly, we had previously showed that reduced osteoprotegerin expression is an independent predictive factor for a poor prognosis in patients with primary colorectal carcinoma [4]. Therefore, osteoprotegerin expression may be a significant predictor of clinical outcomes in patients with colorectal carcinoma with or without liver metastasis.

In conclusion, we demonstrated that reduced osteoprotegerin expression is significantly associated with the extent of colorectal liver metastasis, particularly the multiplicity and bilaterality of metastatic tumors. Moreover, reduced osteoprotegerin expression was independently predictive of the occurrence of recurrent liver metastasis after hepatic resection and reduced overall survival of patients with colorectal liver metastasis. These findings indicate that osteoprotegerin may be a novel predictor of recurrent liver metastasis and a prognostic biomarker in patients with colorectal liver metastasis. We suggest that patients harboring osteoprotegerin-reduced colorectal liver metastasis receive careful monitoring after hepatic resection for colorectal metastasis to enable early detection of potentially resectable recurrent liver metastasis.

## MATERIALS AND METHODS

### Patient and tissue specimens

The 81 patients in this study met the following criteria for hepatic resection with curative intent: 1) medical fitness for major hepatic resection; 2) colorectal liver metastasis that resulted in adequately sized, well-vascularized hepatic remnants after hepatic resection; and 3) no signs of extrahepatic metastases in preoperative imaging studies, including chest radiography, abdominal ultrasonography, abdominopelvic computed tomography, and pelvic magnetic resonance imaging [4, 7, 21]. Only patients whose metastases were resectable on presentation were included. Clinicopathological data, including age, sex, number of metastases, size, and distribution of colorectal liver metastases, histological grade and MSI status of primary colorectal carcinoma, occurrence of recurrent liver metastasis, and follow-up time after hepatic resection were assessed. No patients underwent preoperative neoadjuvant chemotherapy or neoadjuvant concurrent chemoradiation therapy. The protocols for the use of human tissue were approved by the Institutional Review Board of Kangbuk Samsung Hospital, Seoul, Republic of Korea (2015-04-053).

The resected tissues were fixed in 10% neutral-buffered formalin for 24-72 h at room temperature and embedded in paraffin blocks. Each formalin-fixed, paraffin-embedded block was sectioned at 4-μm thickness on a standard rotary microtome, and slices were transferred from a water bath on clean slides and stained with hematoxylin and eosin. Two pathologists independently reviewed the hematoxylin- and eosin-stained slides and selected the most representative slide from each case for immunohistochemical staining.

### Immunohistochemistry

Osteoprotegerin protein expression was assessed by immunohistochemistry using a Bond-maX automatic immunostainer (Leica Biosystems, Buffalo Grove, IL, USA) following the manufacturer's instructions. The general procedure has been previously described [4, 7]: 4-μm sections of formalin-fixed, paraffin-embedded tissue were deparaffinized with Bond Dewax Solution (Leica Biosystems), and an antigen retrieval procedure was performed using Bond Epitope Retrieval Solution (Leica Biosystems) for 30 min at 100°C. Endogenous alkaline phosphatase was quenched using Dual Endogenous Enzyme Block (Dako, Agilent Technologies Inc., Carpinteria, CA, USA) for 5 min, and the samples were incubated with rabbit polyclonal anti-osteoprotegerin antibody (dilution 1:100; Abcam, Cambridge, MA, USA) for 30 min. Slides were then incubated in post-primary AP (Bond Polymer Refine Red Detection, Leica Biosystems) for 20 min, followed by incubation for 30 min in polymer AP (Bond Polymer Refine Red Detection, Leica Biosystems). Sections were then counterstained with hematoxylin. Slides were dehydrated following a standard procedure and sealed with coverslips. To minimize interassay variation, positive and negative control samples were included in each run. The positive control was normal liver tissue, and the negative control was prepared by substituting non-immune serum for the primary antibody; no detectable staining was evident.

### Evaluation of immunohistochemical staining

Immunohistochemical staining was independently analyzed by two pathologists who were blinded to the clinicopathological data and outcomes of the patients. Osteoprotegerin staining intensity was graded as negative (0), weak (1+), moderate (2+), or strong (3+) as previously described [4, 7, 21, 22]. No heterogeneous staining was observed within individual slides, and estimation of the proportion of staining was not required. Disagreements between the two pathologists were resolved by consensus.

### Statistical analysis

A Chi-squared test or Fisher's exact test was performed to determine whether osteoprotegerin expression was associated with clinicopathological characteristics of patients with colorectal liver metastasis. Multivariate logistic regression analysis with a backward stepwise elimination method was used to identify independent significant predictors of recurrent liver metastasis. Univariate and multivariate survival analyses were used to determine the prognostic implications of osteoprotegerin expression. Curves for overall survival were drawn according to the Kaplan-Meier plot, and differences were analyzed by applying the log-rank test for univariate survival analysis. Multivariate survival analysis using the Cox proportional hazard model (95% confidence interval) with a backward stepwise elimination method was also performed. All covariates with statistical significance upon univariate analysis were entered into the multivariate analysis. The least significant covariates were removed from the model by backward stepwise elimination. Statistical analyses were performed using PASW Statistics for Windows (version 18.0; Armonk, NY, USA). Statistical significance was defined as a *P* value less than 0.05.
